# Cybersecurity requirements for medical devices in the EU and US - A comparison and gap analysis of the MDCG 2019–16 and FDA premarket cybersecurity guidance

**DOI:** 10.1016/j.csbj.2025.07.024

**Published:** 2025-07-15

**Authors:** Max Ostermann, Rebecca Mathias, Fatemeh Jahed, Mitchell B. Parker, Florence D. Hudson, William C. Harding, Stephen Gilbert, Oscar Freyer

**Affiliations:** a Else Kröner Fresenius Center for Digital Health, TUD Dresden University of Technology, Dresden, Germany; b Information Security and Compliance, Indiana University Health, Indiana University Health University Hospital, Indianapolis, IN, USA; c Northeast Big Data Innovation Hub, Data Science Institute, Columbia University, FDHint LLC, New York, USA; d College of Graduate and Professional Studies, Trine University, Angola, IN, USA

**Keywords:** Medical devices, Cybersecurity, Regulatory science

## Abstract

The increasing use of connected medical devices has led to substantial cybersecurity challenges, putting patient safety and the integrity of healthcare infrastructures at risk. This study examines regulatory guidance on medical device cybersecurity in the European Union (guidance document of Medical Device Coordination Group MDCG 2019–16 revision 1) and the United States (US Food and Drug Administration Guidance on Cybersecurity) and identifies their strengths and weaknesses. First, the study compares these documents with a baseline requirements framework derived from international standards and best practices, revealing gaps in the thematic areas of “Cryptography,” “Authentication & Access Control,” and “Source Code/Software Development.” Second, the guidance documents were compared with real-world cybersecurity incidents, showing that the current guidance documents would help to mitigate the weaknesses of important vulnerability examples, while recommendations are missing in both guidance documents, but more so in MDCG 2019–16, for the most important weaknesses. In conclusion, both guidance documents are inadequately formulated in certain aspects, have an unclear scope, inconsistent levels of detail, and contain thematic gaps. These gaps could result in manufacturers failing to sufficiently address cybersecurity concerns in their products, thereby creating vulnerabilities. This study highlights the need for future guidance documents to be clearer in scope and to close existing gaps to ultimately allow safer medical devices.

## Graphical Abstract

**Figure:**
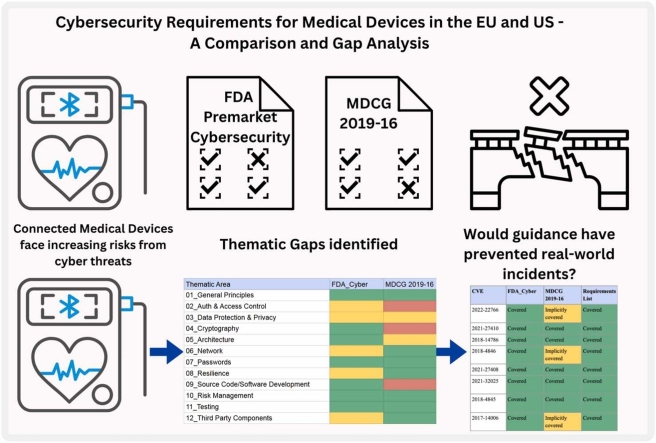

## 1 Introduction

Due to the widespread distribution of software in medical devices (MD) [1], [2] and an increase in cyberattacks on critical healthcare infrastructure [3], [4], cybersecurity has become a significant topic for manufacturers, users, and regulators of MDs. This, on the one hand, is reflected by an increasing number of regulations concerning MD cybersecurity [5] and, on the other hand, by an increase in manufacturers describing cybersecurity features in their documentation, including defensive mechanisms, response systems, or rigorous testing [6].

The aim of MDs is to fulfil their intended purpose effectively and, at the same time, be both clinically and technically safe [7]. However, the use of MDs, in general, also carries risks, which can be caused by many possible aspects of the device. The use of software in MDs connected to the internet adds potentially harmful aspects of cybersecurity risk to the device profile, which could exacerbate existing risks or even introduce new ones [5]. For example, a connected insulin pump could be targeted by malicious actors who could then change the insulin dosage and potentially harm the patient. Furthermore, the pump could be the entry point to a healthcare provider’s system, allowing them to cause further damage to the provider, e.g., through a ransomware attack [8], [9].

Research indicates that connected MDs (cMDs), especially in remote patient monitoring (RPM) or hospital-at-home (HaH) settings, provide a large attack surface to arbitrary actors [10], [11]. This could include attacks on the connected devices themselves, the provider’s infrastructure, hospitals, and patients, and could be conducted via various attack vectors or mechanisms [10]. Such attacks could then, if adequate risk mitigation were not in place, result in patient harm, as described in the example above, where the incorrect dosage of insulin could cause a hyper- or hypoglycaemic coma [11]. Therefore, a device's digital capabilities, its ability to connect to networks, and the implementation of cybersecurity features significantly influence its benefit-risk profile [5].

To prevent, consider or mitigate these risks, a diverse set of standards and best practices has been proposed by international standardisation bodies, national authorities, and researchers [12]. These include, for example, IEC 81001–5–1 about the security of health software and health IT systems [13], the NIST Cybersecurity Framework [14], AAMI TIR57 for the risk management of MDs[15], IEEE 2621 “Standard for Wireless Diabetes Device Security: Information Security Requirements for Connected Diabetes Solutions” for wireless diabetes device security [16], or IEC/TR 60601–4–5, a standard for safety-related technical security specifications [17]. Some of these standards can be categorised as process standards (e.g., IEC 81001–5–1) that provide requirements on how a process should be conducted without providing extensive details on technical aspects. In contrast, product-oriented standards (e.g., IEC/TR 60601–4–5) provide specific requirements, such as security specifications for certain products.

To ensure that manufacturers secure their devices against threats, several jurisdictions have established legal frameworks for the governance of MDs. In the EU, this includes the MD-specific Regulation 2017/745 of the European Parliament and of the Council on medical devices (MDR). This legislation is accompanied by a set of broader non-sector-specific legislation, such as the Cyber Resilience Act (CRA) [18], the Network and Information Systems Directive 2 (NIS2) [19], the EU AI Act [20] and the General Data Protection Regulation (GDPR) [21], which contribute to the EU cybersecurity landscape. They address aspects not directly handled in the MDR, such as network security, data protection and data privacy (GDPR) or the use of artificial intelligence in critical infrastructure (AI Act) to define corresponding requirements. In the US, the structure is similar. High-level requirements for MDs are defined in the Federal Food, Drug, and Cosmetic Act (FD&C Act), while non-sector-specific cybersecurity and data protection aspects are described by other acts.

As the requirements of the legislation are often only high-level, they are further specified by authorities such as the US Food and Drug Administration (FDA) and the EU Medical Device Coordination Group (MDCG) through policies and guidelines [5]. While the FDA is directly involved in the approval process of MDs, the MDCG serves as an expert advisory body formed under Article 103 of the MDR, comprised of representatives from EU member states and chaired by the European Commission. Its primary role is to support the uniform application of the MDR and In Vitro Diagnostic Medical Devices Regulation (IVDR) (Regulation (EU) 2017/746), including the issuance of non-binding guidance documents. The guidance documents issued by these authorities are often based on the aforementioned best practices and standards, supplementing the already existing regulations for MD clearance or approval [5]. Those guidelines and policies define a set of requirements and recommendations and include technical principles, e.g. regarding network security, management aspects such as risk management, and process requirements, e.g. regarding secure development.

Two documents, one from Europe and one from the US, are particularly important for the cybersecurity of medical devices prior to their market release: revision 1 of the “Guidance on Cybersecurity for medical devices” by the MDCG (MDCG 2019–16) for the EU [22], and “Cybersecurity in Medical Devices: Quality System Considerations and Content of Premarket Submissions” (FDA_Cyber) by the US FDA [23]. The goal of these documents is to provide manufacturers and regulators with a clearly defined rule set. However, the extent to which these new regulatory efforts are effective and lead to an improved security situation for MDs has yet to be evaluated. Some researchers and regulatory experts criticised guidance documents as being too superficial and having little practical relevance, thus leading to increased costs without improvement [5].

Literature and academic assessments of the cybersecurity regulatory landscape of MDs is scarce. Prior work has explored the landscape in regard to applicable standards, highlighting the supporting purpose of the MDCG 2019–16 for cybersecurity and the so-called *‘lex specialis’* status of the MDR over MDs, which overrides more general laws and acts such as the CRA or AI Act [24]. In other works, a number of MDCG 2019–16 requirements were loosely mapped to the IEC 80001–5–1 processes [25]. An analysis of a different part of the IEC 80001 analysing the technical controls in IEC 80001–2–2 [26] found it to be an ‘effective baseline’, albeit presenting major options for improvement across areas such as e.g. data recovery, the management of third-party components or confidentiality or integrity of transmitted data. However, academic contributions aiming to propose improvements to the MDCG 2019–16 are also in progress as part of the Horizon Europe Health programme [27], [28].

To evaluate the effectiveness of and potential gaps in current guidelines and offer concrete options to improve the revisions of the MDCG 2019–16 and FDA_Cyber, this study aims to address three research questions. Firstly, do MDCG 2019–16 and FDA_Cyber comprehensively cover relevant cybersecurity aspects and identify potential gaps in themselves? Secondly, could adherence to these frameworks effectively prevent cybersecurity incidents? Thirdly, is the style and structure of guidance set out in MDCG 2019–16 and FDA_Cyber appropriate and helpful regarding the specificity and level of detail?

## 2 Methods

To answer the research questions, a multistep approach was taken. First, a checklist of cybersecurity requirements for MDs was developed based on existing standards and frameworks to provide a baseline for evaluation. Second, MDCG 2019–16 and FDA_Cyber were compared with the checklist and with each other. Third, a set of incidents described in the literature was analysed to identify the weaknesses that led to the incidents. The guidelines and our checklist were mapped against the incidents to assess whether they would have been prevented by following one of the guidelines or our checklist. Fourth, gaps in current guidance documents were described.

### 2.1 Development of the cybersecurity requirements checklist

To assess whether the requirements and recommendations provided in MDCG 2019–16 and FDA_Cyber cover relevant aspects of MD cybersecurity, we first developed a baseline set of cybersecurity requirements. These items of the checklist were extracted from cybersecurity best practices relevant for MDs from recognised entities (Open Worldwide Application Security Project (OWASP) Foundation), international standardisation bodies (International Electrotechnical Commission (IEC) and International Organization for Standardisation (ISO)), national standardisation bodies (AAMI), and national authorities (National Institute of Standards and Technology (NIST)) and Bundesamt für Sicherheit in der Informationstechnologie (BSI). Only high- to medium-level, non-device-specific requirements were included. The requirements were grouped into 12 broader topics following the grouping of the BSI standard (BSI TR-03161) [29] requirements for digital health applications, plus one ‘General’ category. The ‘General’ category was introduced to capture high-level requirements that are overarching and not applicable to individual thematic areas. The initial draft was developed by a cybersecurity expert (author MO) and evaluated by an expert on MD regulation (author OF) and a senior expert on medical device regulation and development (author SG). The requirements were updated based on this feedback. In a second iteration, the checklist’s items were updated with requirements from the recently approved interoperability standard for Internet of Medical Things (IoMT) devices, “IEEE/UL Standard for Clinical Internet of Things (IoT) Data and Device Interoperability with TIPPSS – Trust, Identity, Privacy, Protection, Safety, and Security” (IEEE/UL 2933–2024) [30]. To do so, the “shall” requirements were extracted from IEEE/UL 2933–2024 and analysed for themes missing in the first draft. Following this analysis, eight additional points were included in the checklist. The final checklist was reviewed and approved by all authors.

### 2.2 Comparison and gap analysis of frameworks

For the gap analysis, we included the two most recent cybersecurity guidance documents issued by the FDA and MDCG. Whilst both documents are not legally binding, they communicate the respective authorities' understanding of cybersecurity and are an important point of contact for MD manufacturers. In addition to the premarket guidance included in this study, the FDA has also issued a post-market guidance document. This was excluded from this analysis for three reasons. Firstly, the premarket guidance also contains post-market requirements in sufficient detail. Secondly, the post-market guidance was last updated in 2016 and may, therefore, be out of date. Thirdly, the analysis primarily considers the requirements that manufacturers must fulfil for approval.

To assess the overlap between MDCG 2019–16 and FDA_Cyber, we extracted each requirement from the documents, labelled them, and grouped them under a higher-level theme. MDCG 2019–16 and FDA_Cyber were mapped against the developed set of cybersecurity requirements in a tabular approach to assess the comprehensiveness and quality of these cybersecurity guidance documents. A requirement was considered to be represented if it was explicitly mentioned. This representation was rated for each requirement on a three-point scale from “Sufficiently covered” to “Partially covered” to “Insufficiently covered”. Based on the coverage of individual requirements, the coverage of the higher-level themes was rated on the same scale. Common areas, requirements, and areas of application between the guidance documents and the checklist were identified, and gaps were described. Finally, both documents were compared with each other to identify common areas, areas of application, and divergences.

### 2.3 Incident mapping

To assess whether compliance with MDCG 2019–16 and FDA_Cyber would have a positive effect on the security of MDs, we retrospectively analysed data of cyberattacks on MDs published by the US Cybersecurity & Infrastructure Security Agency (CISA). The CISA publishes reports on attacks on its website and assigns common vulnerabilities and exposures (CVE) codes, linking them to the CVE database. Medical device-related CISA advisories are labelled as ‘ICSMA’ (Industrial Control Systems Medical Advisory). For our analysis, we used the CISA advisories for the top ten vulnerabilities identified by Mejía-Granda et al. (2024) [31]. We exported the data for each example and described each vulnerability. We then analysed whether measures to mitigate the vulnerability were included as requirements in MDCG 2019–16, FDA_Cyber, and the proposed checklist, and thus could have been avoided if the guidance had been followed. The coverage of a vulnerability was rated on a three-point scale, from ‘Covered’ if the guidance explicitly mentioned the corresponding practices or specifications, to ‘Implicitly Covered’ if they are not directly named, but the descriptions indicate that they are covered, to ‘Not Covered’. Any gaps in the guidance documents were identified and described.

## 3 Results

### 3.1 Mapping of guidance documents and the checklist

The initial version of the cybersecurity checklist outlines twelve thematic areas relevant for the secure development and deployment of cMDs, based on the BSI standard introduced in Section 2.1: General Principles, Authentication & Access Control, Data Protection & Privacy, Cryptography, Architecture, Network, Passwords, Resilience, Source Code & Software Development, Risk Management, Testing, and Third-Party Components. In addition to the 12 areas defined in our checklist, FDA_cyber and MDCG 2019–16 contain several recommendations for the submission documentation and user communication. While these are relevant for the approval of MDs, they do not provide process or specification requirements (i.e., a concrete set of steps, technical criteria, or implementation details that guide development and deployment) and, thus, were not included in the checklist. A total of 73 requirements were categorised under these areas. The complete set of requirements, including descriptions and references, is provided in **Appendix I,** Table S1.

**Fig. 1:**
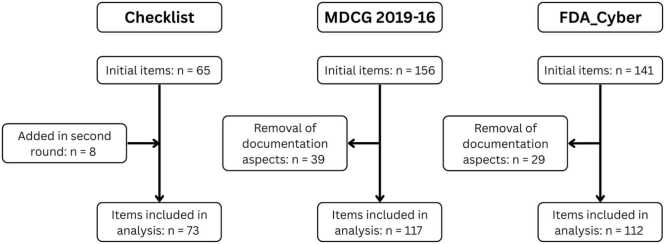
Flowchart of the mapping process.

The requirements and recommendations outlined in the two guidance documents were categorised according to the same categories used for the checklist. For the FDA guidance, a total of 141 requirements were identified, including those related to documentation and user instructions. Similarly, the MDCG guidance includes 156 requirements. A complete mapping of the documents is available in **Appendix I,** Tables S2-S3. For the analysis, items related to documentation aspects were excluded, resulting in 117 MDCG 2019–16 requirements and 112 FDA Cyber requirements. A flowchart illustrating the mapping process is shown in Fig. 1.

### 3.2 Gap analysis of guidance documents

**Fig. 2:**
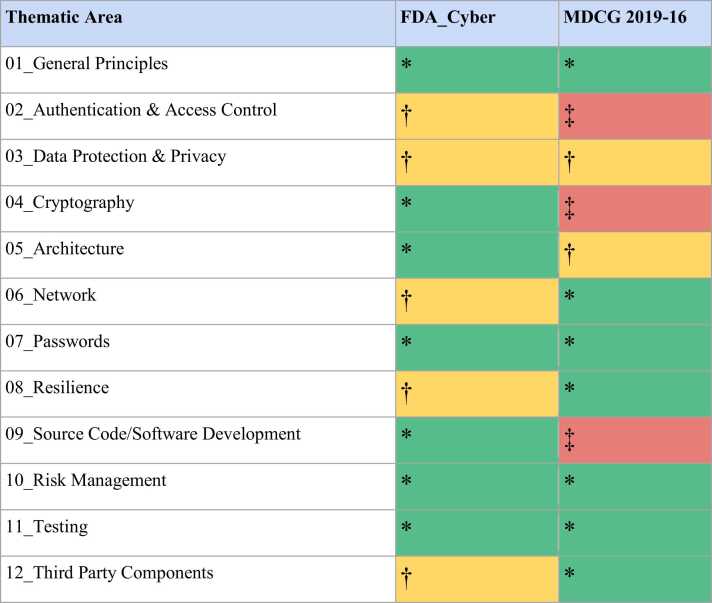
Mapping of MDCG 2019–16 and FDA_Cyber to the requirements list, grouped by higher-level themes. The coverage of the themes is provided on a three-point scale from “Sufficiently covered” (Green, *) to “Partially covered” (Yellow, †) to “Insufficiently covered” (Red, ‡).

The mapping of MDCG 2019–16 and FDA_Cyber shows a varying degree of coverage across the individual thematic areas. General principles were mostly covered; however, individual aspects of the device life cycle (e.g., supply chain security, provisioning, or decommissioning) found no further mention in either guidance. The themes of “Passwords”, “Testing”, and “Risk Management” were covered sufficiently in both guidance documents. FDA_Cyber only partially covered the themes of “Data Protection & Privacy”, “Resilience”, “Authentication & Access Control”, and “Third Party Components”, only mentioning “Networks” as part of the entire MD system. MDCG 2019–16 only partially addresses requirements in the areas of “Data Protection & Privacy” and “Architecture”, with larger gaps remaining in the areas of “Authentication & Access Control”, “Cryptography”, and “Source Code/Software Development”. Fig. 2 shows the coverage of the requirements by category. A table view of the complete gap analysis can be found in **Appendix I,** Table S4.

### 3.3 Vulnerability analysis

Except for three CVEs, the weaknesses and exploits that led to a vulnerability are well covered by measures in the guidance documents. The three CVEs not explicitly covered by MDCG 2019–16 can be traced back to one weakness: common weakness enumeration (CWE)-798, Use of Hard-coded Credentials. While this weakness is considered in the FDA guidance, the MDCG 2019–16 does not name credentials in their no hard-coded passwords requirement.

**Fig. 3:**
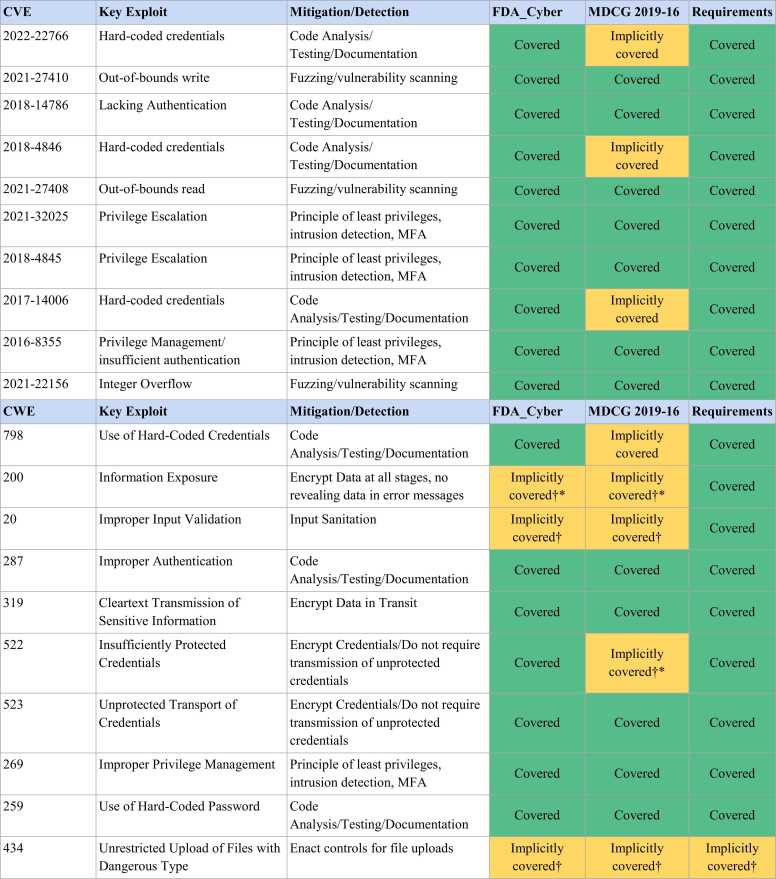
Overview of vulnerabilities and their coverage in guidance documents. The selected CVE examples represent the most impactful medical device weaknesses identified by Mejía-Granda et al. (2024) [20]. The selected CWEs represent the 10 most common weaknesses, according to Bracciale et al. (2023) [32]. The table shows the coverage in the guidance documents and in the requirements list described earlier. *does not offer controls for the examples in CWE-200; † mentioned as part of testing; ǂ predates both guidance documents.

Regarding the weaknesses, the results are more diverse. MDCG 2019–16 covers five CWEs only implicitly: CWE 798 (Use of Hard-Coded Credentials), CWE 200 (Information Exposure), CWE 20 (Improper Input Validation), CWE 522 (Insufficiently Protected Credentials), and CWE 434 (Unrestricted Upload of Files with Dangerous Type). FDA_Cyber performs slightly better, with three CWEs being only implicitly covered: CWE 200 (Information Exposure), CWE 20 (Improper Input Validation), and CWE 434 (Unrestricted Upload of Files with Dangerous Type). Fig. 3 provides an overview of the vulnerabilities and whether these are covered by the guidance document.

## 4 Discussion

### 4.1 Principal results

In our analysis, we identified gaps in current guidance documents, particularly in the areas of “Network” for FDA_Cyber and in the areas of “Cryptography,” “Source Code & Software Development,” and “Authentication & Access Control” for MDCG 2019–16. While the adherence to the principles, requirements, and recommendations of the guidance documents would successfully help to mitigate the weaknesses of the selected CVE examples, recommendations are missing in both guidance documents, but more so in MDCG 2019–16, with regard to the Top 10 Weaknesses (CWEs).

### 4.2 Evaluation of MDCG 2019–16

MDCG 2019–16 provides information on three cybersecurity domains: general principles such as ‘Security by Design’ or ‘Defence in Depth,’ cybersecurity-related processes such as security risk management, and technical specifications.

In order to be compliant with the general requirements of the MDR, the guidance prescribes the application of ‘Secure Design’ and defines it in eight principles. However, the description of these principles is rather superficial, and some principles are only introduced and defined but not properly addressed. This limits the effectiveness of the guidance, as no reference is made to further resources, e.g., OWASP. In addition, the guidance describes a list of security capabilities labelled as ‘indicative,’ which lack sufficient explanation, framing, or justification.

Risk management aspects take up a large part of the guidance and are explained in detail. Except for the link between risk management activities and the quality management system, a requirement according to, for example, IEC 81001–5–1, all important areas are covered. Only the final assessment of remaining cybersecurity risks as part of the benefit-risk analysis remains unclear, so it is uncertain how cybersecurity risks affect the overall benefit-risk profile [5].

In Chapter 3.6, MDCG 2019–16 provides an overview of the minimum IT requirements. It should be emphasised that these requirements should always be considered in the context of the operating environment, a requirement that is also included in the checklist we have proposed. In addition, it is noted that MDs should also be independently cybersecure since requirements for the operating environment are not within the scope of the MDR. Despite this focus, requirements for the MDs themselves remain vague. For example, penetration tests are only mentioned as a recommendation, although they are often treated as a de facto requirement by the Notified Bodies – the organisations designated to assess the conformity of medical devices to their applicable regulations in the EU [33]. Also, important requirements that could prevent known vulnerabilities in MDs are not mentioned, e.g., input sanitisation.

An anomaly in MDCG 2019–16 is the highly detailed requirements for user instructions and documentation, which contrast with the overall vagueness of the technical requirements. It appears somewhat unclear what the purpose of this guidance is. Although the chapters explaining basic principles could serve as a starting point for further activities by manufacturers, the lack of concrete methods, specific requirements, and meaningful references suggests that this guidance may be of limited utility to developers. Additionally, simply following the guidance does not guarantee that the commonly reported weaknesses in MDs are adequately addressed.

### 4.3 Evaluation of FDA_Cyber

The FDA guidance provides a framework for addressing cybersecurity considerations throughout the product lifecycle of cMDs and outlines the requirements and recommendations for documenting this in the premarket submission. The guidance clearly connects device security and safety by defining cybersecurity as a key element of device safety. Additional general principles are also defined for the aspects “Designing for security,” “Transparency,” and “Submission Documentation.”

Similar to MDCG 2019–16, risk management considerations constitute a significant part of the guidance document. The guidance clearly outlines how these considerations should be assessed and offers comprehensive descriptions of activities such as threat modelling and Software Bill of Materials (SBOM), as well as how these could be integrated into the Secure Product Development Framework (SPDF). Notably, the document’s handling of third-party software components and SBOM requirements is more detailed and better articulated than in its EU counterpart. While not mandatory, the SPDF is highly recommended as a set of processes that support MD cybersecurity throughout the devices' lifecycle.

Alongside risk management aspects and overarching principles, the guidance document provides technical recommendations across various domains, including authentication, cryptography, and data integrity. Although these recommendations are suitable at a high level, key aspects such as re-authentication, key protection, or the use of backups are not covered. Ultimately, the guidance clarifies more explicitly than MDCG 2019–16 how to communicate a device's cybersecurity aspects to its users.

### 4.4 Gap analysis of guidance documents

Overall, it can be concluded that both guidance documents address various important aspects and explain fundamental principles of cybersecurity, but they also overlook several technical principles essential for effectively mitigating vulnerabilities. FDA_Cyber performs better than MDCG 2019–16.

A fundamental problem of both guidance documents is that their intended role and the level of detail of their requirements and recommendations are not consistent and thus unclear. Some aspects are only superficially explained or mentioned, while other areas have very specific requirements. This ambiguity regarding the level of detail could be a problem for manufacturers, as some considerations apply to their device, while others do not.

Major gaps in MDCG 2019–16 are in the area of “Authentication & Access Control”, as the document does not consider multi-factor authentication or the establishment of a root of trust through different measures. The consideration of “Data Protection & Privacy” is relatively low, with gaps in the area of data minimisation. It is concerning that there is only superficial guidance on cryptographic principles, such as avoidance of hard-coded credentials. MDCG 2019–16 only explicitly requires the avoidance of hard-coded passwords without considering other credentials, which are part of one of the most common weaknesses in MDs [31], [32]. However, it could be assumed that developers who fulfil the requirements regarding passwords will also follow the implicit requirement for the avoidance of hard-coded credentials in general.

Additionally, considerations of redundancy, attack surface reduction, application of coding best practices, detection of unusual behaviour and purpose, and “No Security through Obscurity” are not appropriately covered. Furthermore, aspects of device management, like provisioning or decommissioning, are only implicitly covered as “secure life-cycle management”. The same applies to manufacturer and supply chain considerations, a topic that recently gained attention following attacks on radio equipment in Lebanon [34]. Related considerations are only implicitly mentioned in MDCG 2019–16 as part of practices 1 and 4.

There are similar gaps in the FDA guidance, where the detection of the change of authentication data, unusual login attempts, or the establishment of a root of trust through different measures are not covered. The document specifies the requirement of secure data at rest, transit, and use only for sensitive data, which is too limiting, as other data could also be of interest to malicious actors. Most important is the lack of attention to network security principles, third-party auditing, detection of unusual behaviour, consent and purpose, and the principle “No Security through Obscurity.”

### 4.5 Implications of real-world incident data

Current research shows that many vulnerabilities and weaknesses exist in on-market MDs, with new issues frequently being discovered [31], [32]. A review of the CVEs related to the ten most impactful MD weaknesses, some of which predate the two guidance documents, indicates that mitigation measures for these are already sufficiently covered in the current guidance. From this, it could be concluded that the mere existence of guidance is insufficient, but that manufacturers must also implement the principles mentioned in the guidance well. A possible approach to encourage stricter adherence could be the exploration of fines in cases of gross misconduct, as is already done in the case of data privacy violations [35]. For the EU, the possibility of penalties for non-compliance with the regulation is outlined in MDR Article 113. The responsible authorities in the member state should consider supplementing existing fines with specific fines for misconduct related to security. However, for these to be an effective tool, enforcement of existing rules is essential, which is not yet sufficiently the case in some MD-specific areas[36].

The findings for the top 10 weaknesses are more varied. Here, too, most are already covered by existing guidance documents, though some CWEs are not explicitly addressed. For MDCG 2019–16, there are five such CWEs, while FDA_Cyber does slightly better with two not explicitly covered CWEs. The CWEs not covered align with the gaps identified in the existing guidance documents.

Some of the CWEs that are covered in the guidance could easily be avoided by careful developers, e.g., hard-coded credentials or privilege issues. It is concerning that these CWEs remain in on-market devices. Other vulnerabilities are harder to detect and to prevent, e.g., out-of-bounds errors. However, careful developers should expect this type of mistake and extensively test the device to detect it.

Although the direct influence of the guidance documents on preventing these CWEs is likely limited, they should address this gap by defining requirements for CWEs that have not yet been adequately covered.

### 4.6 Considerations for future guidance

For further development of guidance documents, it is essential first to clearly define the purpose of the guidance and determine the appropriate level of detail needed for principles or requirements. To avoid inconsistencies, aspects that do not serve this purpose or are not at the agreed level of detail should be excluded. One approach is to formulate general principles and requirements in a high-level guideline, while specific implementation practices and requirements could be detailed in separate device-type-specific guidance documents. This strategy also helps minimise conflicting requirements resulting from the diverse technical profiles of MDs, since cybersecurity requirements for web-based applications, mobile devices, stationary devices, and IoT devices vary considerably. The development of guidance documents should also incorporate the perspectives of multiple stakeholders, including manufacturers and researchers. Checklists like the one developed in this study could serve as a starting point for improved guidance documents.

One issue with defining technical specifications very precisely is that they may quickly become outdated because they no longer reflect the current state of the art. This can be prevented by linking to regularly updated sources or by applying standardisation principles such as those in IEEE 2621, where certain requirements are deliberately left unspecified.

To solve the current problems regarding the document structure, in which requirements are unclear, categorised, listed multiple times, or integrated into running text, the application of the principles of SMART (“Standardised, Machine-readable, Applicable, Readable, Transferrable”) standards could be carried out [37], i.e., to develop SMART guidance. In contrast to current standards and guidance documents, SMART standards are machine-readable, thus simplifying the identification and implementation of requirements and automating them in the future.

### 4.7 Future research

Future research should further investigate what constitutes appropriate and effective guidance documents. The perspectives of relevant stakeholders, such as manufacturers, regulators, healthcare providers, users, or standardisation bodies, should be systematically collected and analysed through methods like DELPHI studies. Such stakeholder engagement will also assist in aligning academically developed theoretical checklists with industry perspectives. The effectiveness of guidance should also be empirically evaluated. To do this, stakeholder perspectives on MD cybersecurity could be gathered, alongside data from devices currently available on the market. The approach proposed in this paper—evaluating publicly available CVEs and CWEs—could form part of this process. Based on these insights, more effective and comprehensive guidance documents can subsequently be developed.

### 4.8 Limitations

This study has several limitations. First, the developed checklist contains ambiguity regarding the level of detail. While some recommendations are high-level, others are more detailed and potentially not appropriate for all kinds of devices. This, however, is also an issue of the guidance documents themselves, as described above. Additionally, there could be inconsistencies regarding the categorisation of some aspects, as they could fall under multiple thematic areas. Second, only MD-specific guidelines were considered in this analysis. In the EU, there are additional regulations related to cybersecurity, including the Cyber Resilience Act [18], the Cybersecurity Act [38], and the NIS Directive [19]. Third, the categorisation of the requirements from the guidance documents to the 12 thematic areas is susceptible to bias as it was based on the judgment of the reviewers. Additionally, requirements could be relevant for more than one area. Fourth, the analysis only retrospectively looked at known vulnerabilities and weaknesses. Thus, the validity regarding new future (and previously unknown) vulnerabilities is limited.

## 5 Conclusions

Cybersecurity vulnerabilities in MDs can jeopardise the safety and effectiveness of these devices and expose patients and healthcare infrastructure to risks. The secure development and deployment of MDs are mandated by regulations and explained in more detail in guidance materials. Our first and third research questions aimed to identify potential gaps in the evaluated guidance documents. We found that these guidance materials are sometimes inadequately formulated, have an unclear scope, inconsistent levels of detail, and contain thematic gaps. These gaps could lead to manufacturers failing to adequately address cybersecurity concerns in their products, creating vulnerabilities. However, in answering research question two, we demonstrated that the most impactful CWEs and CVEs could be mitigated by following the guidance documents, provided that developers also adhere to the aspects that are only implicitly covered. Future guidance documents should be clearer in scope, address existing gaps, and be flexible enough to adapt to rapid developments in the field. Overall, product safety mainly depends on how well manufacturers implement well-established principles, with effective guidelines supporting them and regulatory enforcement identifying and rectifying non-compliance.

## CRediT authorship contribution statement

**Rebecca Mathias:** Writing – review & editing, Data curation. **Max Ostermann:** Writing – review & editing, Writing – original draft, Methodology, Investigation, Formal analysis, Data curation, Conceptualization. **Mitchell B Parker:** Writing – review & editing, Data curation. **Fatemeh Jahed:** Writing – review & editing, Validation. **William C Harding:** Writing – review & editing, Data curation. **Florence D Hudson:** Writing – review & editing, Data curation. **Oscar Freyer:** Writing – review & editing, Writing – original draft, Project administration, Methodology, Formal analysis, Data curation, Conceptualization, Supervision. **Stephen Gilbert:** Writing – review & editing, Writing – original draft, Supervision, Project administration, Methodology, Funding acquisition, Conceptualization.

## Declaration of Competing Interest

The authors declare the following competing interests. SG is an advisory group member of the Ernest & Young-coordinated “Study on Regulatory Governance and Innovation in the field of Medical Devices” conducted on behalf of the Directorate-General for Health and Food Safety of the European Commission. SG has or has had consulting relationships with Una Health GmbH, Lindus Health Ltd, Flo Ltd, Thymia Ltd, FORUM Institut für Management GmbH, High-Tech Gründerfonds Management GmbH, and Ada Health GmbH, and he holds share options in Ada Health GmbH. OF has a leadership role and holds stock in WhalesDontFly GmbH and has had consulting relationships with Prova Health Ltd. MO declares no competing interests. WH declares no conflict of interest relevant to this work. MP declares no conflict of interest relevant to this work. FH declares no conflict of interest relevant to this work. RM declares no conflict of interest relevant to this work. FJ declares no conflict of interest.
