# Supplementary material for: Cybersecurity requirements for medical devices in the EU and US - A comparison and gap analysis of the MDCG 2019–16 and FDA premarket cybersecurity guidance
Source: Comput Struct Biotechnol J. 2025 Jul 15;28:259–66. doi: 10.1016/j.csbj.2025.07.024 (PMC12301760; doi:10.1016/j.csbj.2025.07.024)
Supplement: Supplementary file 1 — Supplementary material [file mmc1.docx]

Cybersecurity Requirements for Medical Devices in the EU and US - A Comparison and Gap Analysis - Appendix I

Max Ostermann^1^, Rebecca Mathias^1^, Fatemeh Jahed^1^, Mitchell B Parker^2^, Florence D Hudson^3^, William C Harding^4^,  Stephen Gilbert^1*^, and Oscar Freyer^1^*^†^

^1^Else Kröner Fresenius Center for Digital Health, TUD Dresden University of Technology, Dresden, Germany

^2^Information Security and Compliance, Indiana University Health, Indiana University Health University Hospital, Indianapolis, IN, USA;

^3^Northeast Big Data Innovation Hub, Data Science Institute, Columbia University, FDHint LLC, New York, USA

^4^College of Graduate and Professional Studies, Trine University, Angola, IN, USA

* Equal contribution as senior author

† Correspondence: oscar.freyer@tu-dresden.de

Table S1. Cybersecurity requirements for medical devices.

| **Code** | **Aspect** | **Description** | **Source** |
| --- | --- | --- | --- |
| **01_General Principles** | |  |  |
| GNRL_1 | Life-Cycle Approach | Security is part of the software development and life cycle. | TR_03161, IEC 81001-5-1, AAMI_TIR57 |
| GNRL_2 | Security by Design (SbD) | The SbD principle shall be considered during the entire development process | ISO 11073-40102:2022; BSI TR-03161-2 "O.Arch_1" |
| GNRL_3 | Security by Default | An application shall automatically be configured in the most secure way | ISO 11073-40102:2022 |
| GNRL_4 | Usable Security | Accept the human-factor to cybersecurity. Ensure security features are user-friendly, adaptable so users don't attempt to circumvent them | NIST SP 800-63B, ISO 9241-210 |
| GNRL_5 | No Security through Obscurity | Rely on well-documented, tested and implemented security measures instead of relying on hidden self-made mechanisms | ANSM R3; BSI TR-03161-2 O.Cryp_2, IEC 81001-5-1 |
| GNRL_6 | Security in Context | Consider the device in its environment, minimisation of physical access ports, physical security | IEEE_2621.2, IEC 81001-5-1 |
| GNRL_7 | Third Party Auditing | Conduct regular audits of third-party components and apply necessary updates promptly | NIST Cybersecurity Framework; IEC 62304 5.1.5 ; BSI TR-03161-2 "O.Arch_1", ANSM R6,R37 |
| GNRL_8 | End-Of-Life | Create a process for securely decommissioning systems and sanitizing data | ANSM, SK, BSI, NIST CSF 2.0 ID.AM-08, IEC 81001-5-1 |
| GNRL_9 | Documentation | The manufacturer should maintain a documentation following industry best practices |  |
| GNRL_10 | Manufacturing / Supply Chain | The manufacturer should consider possible supply chain risks and take actions to mitigate them | IEEE/UL 2933-2024 |
| GNRL_11 | Secure Provisioning | The manufacturer should ensure that provisioning can be done securely in a manner that ensures no leakage of e.g. initial passwords | IEEE/UL 2933-2024 |
| **02_Auth & Access Control** | | | |
| AUTH_1 | Principle of Least Privileges | Every user should only have the least amount of privileges they need to use the application | ISO 11073-40102:2022, IEC 81001-5-1 |
| AUTH_2 | Role Based Access Control | Access control should follow the role based model | ISO 11073-40102:2022 |
| AUTH_3 | Multi-Factor Authentication (MFA) | Implement MFA for all sensitive data access points | TR_03161 |
| AUTH_4 | Session Management | The manufacturer should have a concept for authentication, authorization (role concept) and termination of application sessions. | TR_03161 |
| AUTH_5 | Re-authentication after inactivity or interuptions | Re-authentication after an appropriate period of time during which the application was not actively used or in case of interrupted application. | TR_03161, IEEE_2621.2 |
| AUTH_6 | Access Logging | Enable logging for all access events and regularly review logs | ANSM R29,30 |
| AUTH_7 | Unusual login attempts | Inform the user about unusual login attempts | TR_03161, IEEE_2621.2 |
| AUTH_8 | Root of trust | Signatures and message authentication codes should be verfied by using a hardware-protected root of trust | IEEE_2621.2 |
| **03_Data Protection & Privacy** | | | |
| DATA_1 | Privacy-preserving design | Consideration of the processing of sensitive data in the design phase. | TR_03161 |
| DATA_2 | Minimal access to sensitive data | Dedicated access to encrypted storage or user data through interpreted code only if absolutly necessary. | TR_03161 |
| DATA_3 | Secure Data at Rest | Data is only stored in encrypted form, only decrypted for use, Encryption of locally stored data with a secure Device binding. | NIST CSF2.0 PR.DS, TR_03161 |
| DATA_4 | Secure Data at Transit | Data is only transmitted in encrypted form over secure channels | NIST CSF2.0 PR.DS, TR_03161 |
| DATA_5 | Secure Data at Use | Use of TEE, SMPC or other approaches | NIST CSF2.0 PR.DS, TR_03161 |
| DATA_6 | Third-party data access | No access to sensitive data by third parties. | TR_03161 |
| DATA_7 | Data Minimisation | The manufacturer should follow principles of data minimisation and purpose limitation | TR_03161 |
| DATA_8 | Verifying data validity | Use signatures or message authentication codes to verify validity of data | IEEE_2621.2 |
| **04_Cryptography** | | | |
| CRYP_1 | Strong Cryptographic Algorithms | Use only strong, up-to-date algorithms like AES-256 and RSA-2048 | BSI TR-03161-2 O.Cryp_3,4,5, IEEE_2621.2 |
| CRYP_2 | Key Management | Developers should key management best practices. Key management processes describe the secure generation, storage, distribution, and destruction of keys. | NIST SP 800-57 Part 1 |
| CRYP_3 | Strong Cryptographic Keys | Implement secure key management practices, including rotation and access control | BSI TR-03161-2 O.Arch_3 |
| CRYP_4 | Protection of cryptographic keys | Cryptographic keys should be protected against manipulation. | TR_03161, IEC 81001-5-1 |
| CRYP_5 | No hard-coded keys, credentials or other secrets | Keys should not be hard coded into the source code. | TR_03161 |
| CRYP_6 | Proven Implementations | Only use proven implementations and libraries of crypthographic protocols |  |
| **05_Architecture** | | | |
| ARCH_1 | Secure Update Mechanism | Secure software updates with cryptographic signatures and version controls | ISO 11073-40102:2022, IEC 81001-5-1 |
| ARCH_2 | Central logging system | The application should have a central logging system. | TR_03161 |
| ARCH_3 | Endpoint security | Implementation of security features on all endpoints. | TR_03161, IEC 81001-5-1 |
| ARCH_4 | Management interfaces | Prevent unwanted access via management interfaces. | TR_03161 |
| ARCH_5 | Defense in Depth | The architecture should be designed to provide Defense in Depth | IEC 81001-5-1 |
| ARCH_6 | Unique IDs | Each device should be assigned a unique ID, that can be used to identify a device throughout its life-cycle | IEEE/UL 2933-2024 |
| ARCH_7 | Zero trust architectures | The system architecture should follow a zero trust approach, where other system components are not inherently trusted, but verified. | IEEE/UL 2933-2024 |
| ARCH_8 | Design/Architecture reviews | Architecture should be reviewed on a regular basis | IEC 81001-5-1 |
| 06_Network | | | |
| NETW_1 | Network Security | Secure all network communications with VPNs or encrypted protocols like TLS | ANSM R23-27, NIST SP 800-52 Rev. 2 |
| NETW_2 | Firewalls | Protection of the network by firewalls. | TR_03161 |
| **07_Passwords** | | | |
| PWRD_1 | Strong Password Rules | Implement password complexity and rotation policies aligned with best practices, Apply password policies for complexity and rotation; Maintain cybersecurity usability considerations to prevent unreasonable password requirements (too lenient or too strict) | ANSM R10, TR_03161 |
| **08_Resilience** | | | |
| RESI_1 | Integrity checks of components | The application should detect and prevent a startup in a development/debug environment. | TR_03161 |
| RESI_2 | Redundancy | MDs should integrate redundancy where appropriate (e.g. redundant networking capabilities) | IEEE/UL 2933-2024 |
| RESI_3 | Physical Security | A device should be adequately secured against physical attacks such as tampering. | IEEE/UL 2933-2024 |
| RESI_4 | Fail-Safes | An application should offer a fail-safe mode that ensure a safe state is reached in case of failure or breaches | IEEE/UL 2933-2024 |
| RESI_5 | Measures against reverse engineering | Implement measures against reverse engineering. | TR_03161 |
| **09_Source Code/Software Development** | | | |
| SOUR_1 | Coding Best Practises | Follow secure coding guidelines, such as OWASP or CERT, and conduct regular code reviews | OWASP Secure Coding Practices; CERT Secure Coding, ASNM R37, IEC 81001-5-1 |
| SOUR_2 | Input sanitation | Inputs should be checked before processing. | TR_03161 |
| SOUR_3 | Secure development environment | Activating modern security mechanisms of the development environment. | TR_03161 |
| **10_Risk Management** | | | |
| RISK_1 | Continuous Monitoring | Use appropriate level of real-time monitoring tools to detect and alert on potential security events; the monitoring should include publicly available incident and vulnerability databases | NIST CSF2.0 DE, ID.IM-02, IEC 81001-5-1, AAMI_TIR57, TR_03161 |
| RISK_2 | Incident Response Plan | Develop and test a formal incident response plan | ISO/IEC 27035, NIST CSF2.0 RS, ID.IM-04, IEC 81001-5-1, TR_03161 |
| RISK_3 | Backups | An application should integrate backup functionality to minimize data loss in cases of security breaches or device issues. | IEEE/UL 2933-2024 |
| RISK_4 | Threat Modeling | Create a comprehensive threat model identifying potential threats, vulnerabilities, assets, and adverse impacts | https://owasp.org/www-community/Threat_Modeling, NIST SP 800-30; IEC 80001-1, IEC 81001-5-1, AAMI_TIR57 |
| RISK_5 | Check vulnerability databases | Regularly assess the application against OWASP Top 10 vulnerabilities and CWE weaknesses | ANSM R37 |
| RISK_6 | User instructions | The manufacturer should inform the user about the safe handling of the product | TR_03161, IEC 81001-5-1 |
| RISK_7 | Quality Management System | Security activities should be performed on the basis of a documented quality management system | IEC 81001-5-1 |
| RISK_8 | Security Training | Security training should be provided to the users if needed and to the manufacturers staff | IEC 81001-5-1 |
| RISK_9 | Security Risk Management | Security Risk Management should be implemented, e.g. by following ISO 14971, and should include threat modeling and risk estimation for each vulnerability and threat combination as well al a review of implemented security measures. | IEC 81001-5-1, AAMI_TIR57 |
| RISK_10 | User reporting | Users should be able to report security issues easily. | TR_03161, IEC 81001-5-1 |
| RISK_11 | Reporting of security-related issues to authorities | The manufacturer should report security related issues to authorities | IEC 81001-5-1 |
| RISK_12 | Benefit Risk analysis | The benefit-risk analysis should consider cybersecurity-related risks. | AAMI_TIR57 |
| **11_Testing** | | | |
| TEST_1 | Penetration testing | Conduct regular penetration testing to identify and mitigate vulnerabilities by third parties and internally | BSI TR-03161-2 , AAMI_TIR57, IEC 81001-5-1 |
| TEST_2 | Security requirements testing | Internal tests wether security requirements are met | IEC 81001-5-1, AAMI_TIR57 |
| TEST_3 | Threat mitigation testing | Test the ability and effectivness against threats identified in the threat modeling | IEC 81001-5-1, AAMI_TIR57 |
| TEST_4 | Vulnerability testing | The application should be regularly tested/assessed for vulnerabilities | IEC 81001-5-1 |
| TEST_5 | Static code analysis | Use of tools for static code analysis of the source code. | TR_03161, IEC 81001-5-1 |
| **12_Third Party Components** | | | |
| THRD_1 | Limitation of third-party componenets | Use only required third-party software and componenets | TR_03161 |
| THRD_2 | Maintenance of third-party components | The manufacturer should check the maintenance of third-party software/components used over the devices life cycle. | TR_03161 |
| THRD_3 | (S)BOM | The manufacturer should provide a centralized and complete list of dependencies from third party software. | TR_03161 |
| THRD_4 | Secure third-party components/software | Third-party suppliers should perform security life cycle activities | IEC 81001-5-1 |

Table S2. Mapping of FDA cybersecurity requirements for medical devices.

| Area | Aspect | Description |
| --- | --- | --- |
| General | | |
|  | Risk minimisation regarding known vulnerabilites | A device should be designed to eliminate or mitigate known vulnerabilities. Vulnerabilities identified in Cybersecurity and Infrastructure Security Agency (CISA) Known Exploited Vulnerabilities Catalog should be designed out of the device |
|  | Cybersecurity Design | FDA recommends that device manufacturers’ design processes include design inputs for cybersecurity controls. |
|  | Security in Context | Cybersecurity activities and device specifications should consider the intended use and the operating environment |
|  | Deny by default | Design devices to “deny by default” (i.e., that which is not expressly permitted by a device is denied by default) |
|  | Security by design/default | Design devices such that the potential impact of vulnerabilities is limited by specifying a secure configuration. Secure configurations may include endpoint protections, such as anti-malware, firewall/firewall rules, allow-listing, defining security event parameters, logging parameters, physical security detection, and/or HIDS/HIPS. |
| Auth | | |
|  | Use appropriate authentication | Use appropriate user authentication (e.g., multi-factor authentication to permit privileged device access to system administrators, service technicians, or maintenance personnel, among others, as needed) |
|  | Authentication and authorisation for critical functionality | Require authentication, and authorization in certain instances, before permitting software or firmware updates, including those updates affecting the operating system, applications, and anti-malware functionality |
|  | Authentication failures | Consider how the device and/or system should respond in event of authentication failure(s) |
|  | Principle of Least Privileges | the principle of least privileges hould be applied |
|  | Access limitation | Limit authorized access to devices through the authentication of users |
|  | Automatic time out | Use automatic timed methods to terminate sessions within the medical device system where appropriate for the use environment |
| Data | | |
|  | Data Accessibility | cybersecurity controls should not be intended to prohibit a user from accessing their device data. |
|  | Anti replay measures/Data validity | Implement anti-replay measures in critical communications such as potentially harmful commands |
|  | Verify Data Authenticity | Provide mechanisms for verifying the authenticity of information originating from the device, such as telemetry |
|  | Data integrity | Verify the integrity of all incoming data, ensuring that it is not modified in transit or at rest |
|  | Data validity | Validate that all data originating from external sources is well-formed and compliant with the expected protocol or specification. Additionally, as appropriate, validate data ranges to ensure they fall within safe limits |
|  | Data integrits protection | Protect the integrity of data necessary to ensure the safety and effectiveness of the device, e.g., critical configuration settings such as energy output. |
|  | Secure Sensitive Data at Rest | Sensitive Data at transit should be encrypted |
|  | Secure Sensitive Data at Transit | Sensitive Data at rest should be encrypted |
|  | Runtime code integrity verification | Use industry-accepted best practices to maintain and verify integrity of code while it is being executed on the device |
|  | Recovery of device configuration | Design devices to provide methods for retention and recovery of trusted default device configuration by an authenticated, authorized user |
| Cryptography | | |
|  | Strong Cryptographic Algorithms | Select industry-standard cryptographic algorithms and protocols, and select appropriate key generation, distribution, management and protection, as well as robust nonce mechanisms. |
|  | Strong Cryptographic Algorithms | Use current NIST recommended standards for cryptography or equivalent-strength cryptographic protection |
|  | Strong Cryptographic Algorithms | Implement cryptographic protocols that permit negotiated parameters/versions such that the most recent, secure configurations are used, unless otherwise necessary. |
|  | Strong Cryptographic Algorithms | Use cryptographically strong authentication schemes |
|  | Hardware-based security | Hardware-based security solutions should be considered and employed when possible |
|  | Avoid CRC | Do not rely on cyclic redundancy checks (CRCs) as security controls |
| Architecture | | |
|  | Security by design | Implementation of security controls should be applied across the system architecture |
|  | Authentication of external connections | Authenticate external connections at a frequency commensurate with the associated risks |
|  | Defense in depth | Design a system architecture and implement security controls to prevent a situation where the full compromise of any single device can result in the ability to reveal keys for other devices. |
|  | Secure Update Mechanism | Digitally and physically Authenticate firmware, software, and devices |
|  | Secure Update Mechanism | Allow installation of cryptographically authenticated firmware and software updates, and do not allow installation where such cryptographic authentication either is absent or fails |
|  | Validate software prior execution | Ensure that the authenticity of software, firmware, and configuration are validated prior to execution |
|  | Forensic evidence/Logging system | Ensure the design enables forensic evidence capture. The design should include mechanisms to securely create and store log files off the device to track security events. |
|  | Updateability | Design devices to anticipate the need for software and firmware patches and updates to address future cybersecurity vulnerabilities |
|  | Secure Update Mechanism | Consider update process reliability and how update process works in event of communication interruption or failure |
|  | Security updates independent of cycle | Consider cybersecurity patches and updates that are independent of regular feature update cycles |
|  | Ensure application of updates | Implement processes, technologies, security architectures, and exercises to facilitate the rapid verification, validation, and distribution of patches and updates. |
|  | Trusted update channels | Implement a secure process and mechanism for providing validated software updates and patches for users. |
|  | Cybersecurity interoperability | Cybersecurity controls should be used as a means to allow for the safe and effective exchange and use of information with other systems |
|  | Endpoint security | Design devices such that they may integrate and/or leverage antivirus/anti-malware protection capabilities. |
|  | Permit tracking and control of software changes | Design devices to enable software configuration management and permit tracking and control of software changes to be electronically obtainable (i.e., machine readable) by authorized users. |
|  | Forbid downgrades | Do not allow downgrades, or version rollbacks, unless absolutely necessary for safety reasons, and log and document the event |
| Network | | |
|  |  |  |
| Passwords | | |
|  | No hardcoded passwords | Do not use passwords that are hardcoded |
|  | Strong password rules | Do not use passwords that are default, easily guessed, or easily compromised |
| Resilience | | |
|  | Disable debug ports | Disable or otherwise restrict unauthorized access to all test and debug ports prior to delivering products |
|  | Physical security | Employ tamper evident seals on device enclosures and their sensitive communication ports to help verify physical integrity |
|  | Resilience of sub systems and components | Design devices to specify the level of resilience, or independent ability to function, that any component of the medical device system possesses when its communication capabilities with the rest of the medical device system are disrupted, including disruption of significant duration |
| Source Code/Software Development | | |
|  | Coding best practices | manufacturers must implement a development processes that account for and address software risks throughout the design and development process as part of design controls |
|  | Secure Product Development Framework | Develop and use a Secure Product Development Framework (SPDF) |
|  | control of device source code | device manufacturers should establish and maintain custodial control of device source code |
|  | Secure development environment | Preserve and maintain full build environments and virtual machines, regression test suites, engineering development kits, emulators, debuggers, and other related tools that were used to develop and test the original product to ensure updates and patches may be applied safely and in a timely manner |
|  | Incident Detection/Security by design | Implement design features that allow for security compromises and suspected compromise attempts to be detected, recognized, logged, timed, and acted upon during normal use |
|  | Vulnerability Detection/Security by design | Design devices to facilitate the performance of variant analyses such that the same vulnerabilities can be identified across device models and product lines. |
|  | Segmentation of critical functions | Implement features that protect critical functionality and data, even when the device has been partially compromised |
|  | Security by Design | Design devices to be resilient to possible cyber incident scenarios such as network outages, Denial of Service, excessive bandwidth usage by other products, disrupted quality of service (QoS), and/or excessive jitter |
|  | Input Sanitation | Design devices to be resilient to possible noise items |
| Risk Management | | |
|  | Security Risk Management | software device manufacturers may need to establish cybersecurity risk management and validation processes |
|  | Security Risk Management | Manufacturers should ensure they have appropriate resources to identify, assess, and mitigate cybersecurity vulnerabilities as they are identified throughout the supported device lifecycle |
|  | Security Risk Management | These processes should also ensure that risk control measures for one type of risk assessment do not inadvertently introduce new risks in the other. |
|  | Life-Cycle Approach | Security risk management should be an integrated part of a manufacturer’s entire quality system, addressed throughout the TPLC. |
|  | Security Risk Management Plan | Generate a security risk management plan, e.g. in accordance with AAMI TIR 57 |
|  | Risk assessment | FDA recommends that device manufacturers conduct both a safety risk assessment and a separate, accompanying security risk assessment to ensure a more comprehensive identification and management of patient safety risks. |
|  | Risk assessment | when any known vulnerabilities are only partially mitigated or unmitigated by the device design, they should be assessed as reasonably foreseeable risks in the risk assessment and be assessed for additional control measures or risk transfer35 to the user/operator, or, if necessary, the patient. |
|  | Risk assessment | A cybersecurity risk assessment should be conducted which focusses on exploitabilities |
|  | Risk assessment | The cybersecurity risk assessment is non-probalistic |
|  | Risk assessment | security risk assessment processes focus on exploitability, or the ability to exploit vulnerabilities present within a device and/or system. |
|  | Risk assessment | manufacturers should assess identified risks according to the level of risk posed from the device and the system in which it operates. |
|  | Risk assessment | If post market exploits are not applicable to unreleased software, a premarket exploitability assessment could either assume a worstcase assessment and implement appropriate controls, or provide a justification for a reasonable exploitability assessment of the risk throughout the TPLC and how the risk is controlled. |
|  | Risk assessment | Acceptance criteria for cybersecurity risks should carefully consider the TPLC of the medical device system |
|  | Risk assessment | known vulnerabilities should be assessed as reasonably foreseeable risks |
|  | Risk assessment TPLC | The cybersecurity risk assessment should consider the TPLC |
|  | Risk assessment | Security risk assessments that include analyses and considerations of cybersecurity risks that may exist in or be introduced by third-party software and the software supply chain could conducted |
|  | Risk assessment | For components with known vulnerabilities, device manufacturers should provide Details of applicable safety and security risk controls to address the vulnerability. |
|  | Risk assessment | the assessment for impacts to safety and effectiveness may include an assessment for the potential security impacts of anomalies including consideration of any present Common Weakness Enumeration (CWE) categories |
|  | Risk Transfer | Any risks transferred to the user should be detailed and considered for inclusion as tasks during usability testing |
|  | Risk Transfer | Risk transfer, if appropriate, should only occur when all relevant risk information is known, assessed, and appropriately communicated to users |
|  | Threat Modeling | Conduct Threat Modeling |
|  | Threat Modeling | The threat model should Identify medical device system risks and mitigations as well as inform the pre- and post-mitigation risks considered as part of the cybersecurity risk assessment; |
|  | Threat Modeling | The threat model should State any assumptions about the medical device system or environment of use |
|  | Threat Modeling | The threat model should Capture cybersecurity risks introduced through the supply chain, manufacturing, deployment, interoperation with other devices, maintenance/update activities, and decommission activities |
|  | Risk Measures and Metrics | measures and metrics for the assessment over the TPLC should be defined and measured |
|  | Risk Measures and Metrics | Risk measures and metrics could include Percentage of identified vulnerabilities that are updated or patched (defect density); |
|  | Risk Measures and Metrics | Risk measures and metrics could include Duration from vulnerability identification to when it is updated or patched |
|  | Risk Measures and Metrics | Risk measures and metrics could include Duration from when an update or patch is available to complete implementation in devices deployed in the field, to the extent known. |
|  | Check vulnerability databases | Cybersecurity management plans should include Identify and address vulnerabilities identified in CISA Known Exploited Vulnerabilities Catalog |
|  | Known Vulnerabilites | For each known vulnerability, manufacturers should describe how the vulnerabilities were discovered to demonstrate whether the assessment methods were sufficiently robust. |
|  | Known Vulnerabilites | For components with known vulnerabilities, device manufacturers should provide A safety and security risk assessment of each known vulnerability |
|  | Reporting of security-related issues | Cybersecurity management plans should include Description of their coordinated vulnerability disclosure process |
|  | User notification in case of incidents | Design devices to notify users when malfunctions or anomalous device behavior, including those potentially related to a cybersecurity breach, are detected. |
| Testing | | |
|  | Testing | FDA recommends verification and validation include sufficient testing performed by the manufacturer on the cybersecurity of the medical device system through which the manufacturer verifies and validates their inputs and outputs |
|  | Security requirements testing | Security requirements testing |
|  | Threat mitigation testing | Threat Mitigation testing |
|  | Vulnerability testing | Vulnerability testing (e.g., Fuzz Testing, Static and dynamic code analysis, attack surface analysis) |
|  | Penetration testing | Penetration testing |
|  | Static code analysis/Code Review | Carefully design and review all code that handles the parsing of external data using automated (e.g., static and dynamic analyses) and manual (i.e., code review) methods |
|  | Life Cycle Approach | FDA recommends that cybersecurity testing should occur throughout the TPLC |
| Third Party Components | | |
|  | Risk management | Third party software components should be assessed for cybersecurity risks |
|  | Risk management | Third party software components risks should be addressed or mitigated |
|  | Risk management | security risks of the third party software components should become factors of the overall medical device system risk management processes and documentation |
|  | Third-party update | manufacturers should include plans for how third-party software components could be updated or replaced if support ends or other software issues arise |
|  | SBOM | An SBOM should be provided and maintained that includes the device manufacturer-developed components and thirdparty components |
|  | SBOM | The SBOM should be machine readable, and contain the minimum requirements of the National Telecommunications and Information Administration (NTIA) Multistakeholder Process, the the level of support, and the end of support date |
|  | Third-party licencies | Maintain necessary third-party licenses throughout the supported lifespan of the device. |
|  | Third-party EoS | Develop contingency plans for the possibility that a third-party company goes out of business or stops supporting a licensed product. |
| User instructions/Communication | |  |
|  | User Cybersecurity Information | Manufacturers should inform users of relevant security information |
|  | Transparency of network ports | Provision of a list of network ports and other interfaces that are expected to receive and/or send data to users. |
|  | User instruction | User should be provided with instructions that allow them to manage risks associated with the software components, including known vulnerabilities, configuration specifications, and other relevant security and risk management considerations |
|  | User instruction | Sufficiently detailed diagrams for users that allow recommended cybersecurity controls to be implemented |
|  | User instruction | Specific guidance to users regarding supporting infrastructure requirements so that the device can operate as intended |
|  | Update instructions | A description of systematic procedures for users to download version-identifiable manufacturer-authorized software and firmware, including a description of how users will know when software is available. |
|  | User information | FDA recommends that manufacturers establish a plan for how they will identify and communicate to users vulnerabilities that are identified after releasing the device |
| Documentation | | |
|  | Documentation | Manufacturers should submit documentation in their premarket submissions demonstrating that the security controls for the categories above, and further detailed in the recommendations in Appendix 1, have (1) been implemented, and (2) been tested in order to validate that they were effectively implemented |
|  | Security in context | Device instructions and product specifications related to recommended cybersecurity controls appropriate for the intended use environment |
|  | Labeling Recommendation | A description of how the design enables the device to respond when anomalous conditions are detected |
|  | Labeling Recommendation | A high-level description of the device features that protect critical functionality |
|  | Labeling Recommendation | A description of backup and restore features and procedures to restore authenticated configurations. |
|  | Labeling Recommendation | A description of methods for retention and recovery of device configuration by an authenticated authorized user. |
|  | Labeling Recommendation | A description of the secure configuration of shipped devices, instructions for userconfigurable changes, and identification of user-configurable changes that could increase security risk for the medical device system |
|  | Labeling Recommendation | Where appropriate for the intended use environment, a description of how forensic evidence is captured, including but not limited to any log files kept for a security event. |
|  | Labeling Recommendation | Information, if known or anticipated, concerning device cybersecurity (including components) end of support and end of life. |
|  | Labeling Recommendation | Information on securely decommissioning devices by sanitizing the product of sensitive, confidential, and proprietary data and software. |
|  | security risk management reports | include security risk management reports in submission |
|  | security risk management reports | The security risk management report should include the documentation elements for the system threat modeling, cybersecurity risk assessment, Software Bill of Materials (SBOM), component support information, vulnerability assessments, and unresolved anomaly assessment(s) |
|  | security risk management reports | the security risk management report should Summarize the risk evaluation methods and processes |
|  | security risk management reports | the security risk management report should Detail the residual risk conclusion from the security risk assessment, |
|  | security risk management reports | the security risk management report should Detail the risk mitigation activities undertaken |
|  | security risk management reports | the security risk management report should Provide traceability between the threat model, cybersecurity risk assessment, SBOM, and testing documentation |
|  | Threat model documentation | Provide Threat modeling documentation |
|  | Update of documentation | manufacturers should update their security risk management documentation as new information becomes available |
|  | Update of documentation | The risk management documentation should account for all versions of the device |
|  | Security architecture views | Different security architecture views should be provided, at a minimum Global System View; • Multi-Patient Harm View; • Updateability/Patchability View; and • Security Use Case View(s) |
|  | Security architecture views | The security architecture views should Identify security-relevant medical device system elements and their interfaces |
|  | Security architecture views | The security architecture views should Establish traceability of architecture elements to user and medical device system security requirements. |
|  | Security architecture views | FDA recommends that manufacturers develop and maintain security architecture view documentation as a part of the process for the design, development, and maintenance of the medical device system |
|  | Cybersecurity Management Plan | Cybersecurity management plans should include Personnel responsible |
|  | Cybersecurity Management Plan | Cybersecurity management plans should include Sources, methods, and frequency for monitoring and identifying vulnerabilities |
|  | Cybersecurity management plan | Cybersecurity management plans should include Timeline to develop and release patches |
|  | Cybersecurity management plan | Cybersecurity management plans should include Update processes |
|  | Cybersecurity management plan | Cybersecurity management plans should include Patching capability |
|  | Cybersecurity management plan | Cybersecurity management plans should include Description of how the manufacturer intends to communicate forthcoming remediations, patches, and updates to customers |

Table S3. Mapping of MDCG 2019-16 cybersecurity requirements for medical devices.

| Area | Aspect | Description |
| --- | --- | --- |
| General | | |
|  | Third Party Auditing | Auditability that supports non-repudiation |
|  | Security in Context | Assess reasonable level of security for the operating environment |
|  | Security by Design (SbD) | the prodcut should be Secure by design |
|  | Life-Cylce Approach | The PMS system should update the design and manufacturing information, the instructions for use and the labelling; |
|  | Security in Context | Establish appropriate security measures for the use of mobile devices and teleworking. |
|  | Life-Cylce Approach | Security, safety and effectiveness should be considered over the devices life cycle |
|  | Security Capabilities for MD | Audit Controls |
|  | Security Capabilities for MD | Configuration of Security Features |
|  | Security Capabilities for MD | Security and Privacy Guides |
|  | Security in Context | operating environment must provide physical security, e.g., through authenticated physical access, segregation, access policy |
|  | Life-Cylce Approach | The post-market cybersecurity surveillance program should include sharing and dissemination of cybersecurity information and knowledge of cybersecurity vulnerabilities and threats across multiple sectors |
|  | Security in Context | Consider Intended use |
|  | Security in Context | Consider operational environment |
|  | Security in Context | Good physical security |
|  | Security by default | Secure configuration of the system at integration |
|  | Security in Context | The operator is responsible for a secure operational environment |
|  | Security in context | Interaction between software and the IT environment |
|  | Regulatory Landscape | The operator must be in line with national and EU regulations |
|  | Security Capabilities for MD | Personal Data Integrity and Authenticity |
|  | Security in context | Physical Locks |
|  | Security in context | Manufacturer should determine the minimum requirements for their devices operating environment |
|  | Security Capabilities for MD | Appropriate security controls |
|  | Security in context | measures should be implemented at the operator site in a time appropriate to the security and safety risk |
| Auth | | |
|  | Access Management | Methods for authentication and authorisation should be appropriate to the device |
|  | Session Management | Automatic Logoff |
|  | Access Management | Emergency Access |
|  | Access Management | Person Authentication |
|  | Session Management | Session management measures |
|  | Principle of Least Privileges | Apply the principle of least privilege to user workstations and connected devices. o Least privileges must also take into account data minimisation per role. |
|  | Role Based Access Control | Access control measures (e.g. role based) |
|  | Network Access control | Network access controls, such as segmentation |
|  | Access Management | User access management |
|  | Unauthorised access | Protection against Unauthorised access |
| Data | | |
|  | Personal Data | Personal Data De-Identification |
|  | Backup/Recovery | Data Backup and Disaster Recovery |
|  | Personal Data | Personal Data Storage Confidentiality |
|  | Secure Data at Transit | Transmission Confidentiality |
|  | Secure Data at Transit | Transmission Integrity |
|  | Secure Data at Transit | Data encryption |
|  | Data inegrity | Data integrity should be ensured e.g. through hashing, integrity checks. |
|  | Backup/Recovery | Implement data recovery mechanisms to restore data from critical systems |
| Cryptography | | |
|  |  |  |
| Architecture | | |
|  | Updatability | General patch management practices that ensure timely security patch updates |
|  | Defense in Depth | Apply Defense in depth |
|  | Updatability | Cybersecurity Product Upgrades |
|  | Security interoperability | Interoperability and compatibility with other devices or products |
|  | Security of devices | medical device should be as autonomous as possible in terms of IT security and sole reliance on the existence of any IT security requirements on the operating environment should be kept to a minimum |
|  | Protection against arbitrary code execution | Memory protection measures to block arbitrary code execution |
|  | Security in Context/Security Interoperability | Elements of the operating environment interacting with (e.g. other devices) or required for the operation of medical devices (e.g. OS) should ensure interoperability and shall not impair the specified performance of the medical device |
|  | Security by Design | Operating system hardening and application whitelisting |
|  | Security Interoperability | Compatibility of medical device management software with security solutions that counter malicious code |
|  | Secure Update Mechanism | Provisions to ensure integrity/validation of software updates and security patches |
|  | Endpoint Security | Antivirus / anti-malware software |
|  | Security requirements | Conduct a Specification of security requirements to identify required security capabilities for the device |
| Network | | |
|  | Firewalls | Protection of devices with firewalla |
|  | Control and security of network traffic | operating environment must provide control and security of network traffic |
|  | Control and security of network traffic | Network segmentation |
|  | Control and security of network traffic | Traffic filtering |
|  | Segmentation | Partitioning mechanisms and network / traffic segmentation |
|  | Workstation Security | Application whitelisting / system hardening |
|  | Secure network environment | Node Authentication |
| Passwords | | |
|  | Strong Password Rules | Use sufficiently complex passwords |
| Resilience | | |
|  | Integrity check of end device | Software integrity checks and device authentication mechanisms |
| Source Code/Software Development | | |
|  |  |  |
| Risk Management | | |
|  | Risk acceptance | security risk has to be reduced to an acceptable level |
|  | Security risk management | Risk management should consider reasonably foreseeable misuse |
|  | Notifications | Notification of the manufacturer in case of events |
|  | Security risk management | Management of security-related issues |
|  | Security risk management | Security risk management is part of the process of the general risk management process |
|  | Safety risk management | Security risks or controls with safety impact should be included in the safety risk assessment |
|  | Security risk management | Safety risks or controls that have a security impact should be included in the security risk assessment |
|  | Safety risk management | The safety risk asssessment should list generic security related hazards (e.g., denial of service) without detailing every possible security attack vector |
|  | Threat Modeling | Conduct Threat modelling to identifiy device vulnerabilites |
|  | Vulnerability identification | Assess the releveance vulnerabilites, e.g., their risk, how they affect safety, etc. |
|  | Benefit-Risk Analysis | For security risks, an overall Benefit Risk Analysis is to be executed based on the intended use and possible safety and performance impact |
|  | PMS | Post-market surveillance should include Security incidents directly related to medical device software |
|  | PMS | Post-market surveillance should include Security Vulnerabilities that are related to the medical device hardware/software and the 3rd party hardware/software used with the medical device. |
|  | PMS | Post-market surveillance should include Changes in the threat landscape, including interoperability aspects |
|  | Life-cycle approach | The manufacturer should evaluate the information thus gathered, evaluate the associated security and safety risk and take appropriate measures that control the risk associated with such security incidents or vulnerabilities |
|  | Response Plan | Documented action plan for the user to follow in case of an alert message |
|  | PMS | The post-market cybersecurity surveillance program should include vulnerability remediation |
|  | PMS | The post-market cybersecurity surveillance program should include incident response |
|  | PMS | The PMS system should update the benefit-risk determination and to improve the risk management; |
|  | Notify Authorities | Serious incidents and field safety corrective actions must be reported to the competent authority |
|  | Notify Authorities | Incidents that have cybersecurity related incident root causes are subject to Trend Reporting |
|  | Risk Assessment | Conduct a risk assessment and impact assessment. Introduction of medical devices in the environment should be subject to such a risk assessment. |
|  | Security in context | The Health Information System (HIS) must be able to monitor the correct operation of the equipment. o Monitor device behaviour in the context of medical workflows. |
|  | Response Plan/Life-cycle approach | Investigate major incidents and review actions taken to mitigate and reduce time to react to future occurrences. |
|  | Response Plan | Develop a disaster recovery plan, taking into account the minimum recovery requirements. |
|  | Risk Assessment | IT security requirement for the operating environment should be based on the risk assessment |
|  | Life Cycle Approach | The post-market cybersecurity surveillance program should include operation of the device in the intended environment |
|  | cyber smart behavior | Users are encouraged to employ cyber smart behavior |
|  | Benefit-Risk Analysis | Risks of to weak and to restricitive security |
|  | Training | Security awareness training |
|  | Risk Assessment | The exploitation of unknown vulnerabilities should be considered as reasonably foreseeable misuse |
|  | Training | Provide required documentation and training |
|  | Patchability | Provide support for patching and security incident handling |
|  | Risk Assessment | include security issues in the risk assessment |
|  | Security risk management | Implement Security management |
|  | Security requriements | When determining security capabilities, the manufacturer should demonstrate for each security measure that not only the goals of safety and effectiveness are maintained with the implementation of a specific capability, but also performance requirements and the existing risk control measures remain effective as specified |
|  | Device catalogue | Catalogue assets in an inventory of all medical devices, servers and workstations. |
|  | Risk Control Measure | Quick fixes, e.g. network configuration changes |
|  | PMS | A Post-market surveillance system shoiuld be put in place |
|  | PMS | The PMS system should update the clinical evaluation |
|  | Security Policies | A set of baseline IT security policies should be defined, approved by management and communicated to employees and relevant external parties, including roles, password policy, etc. |
| Testing | | |
|  | Testing | Security verification and validation testing should be used to ensure that security requirements are met |
|  | Testing | To allow security verification and validation, testing should be conducted |
|  | Security requirements testing | To allow security verification and validation, testing should be conducted |
|  | Vulnerability testing | fuzz testing |
|  | Vulnerability testing | vulnerability scanning |
|  | Penetration testing | penetration testing |
|  | Static code analysis | Use tools for secure code analysis |
|  | Testing of updates | security updates and security patches ashould be tested for regressions and made available to product users in a timely manner |
|  | Static code analysis | Use tools that scan for open source code and libraries used in the product, to identify components with known issues |
| Third Party Components | | |
|  | Limitation of third-party componenets | Install only software programmes necessary for the intended use of the operating environment. |
|  | Supervision of third-party components | Modification of a medical device, e.g. the installation or enabling of third-party software including software patching, should always be under explicit published guidance of the manufacturer. |
|  | Trustworthy third-party-components | Exclusive use of genuine software and ban of all illegitimate software and applications |
|  | EoL of Third Party components | Avoid the use of End of life third-party components and devices on the operating environment, where possible take additional measures such as network isolation. |
|  | Changes of Third Party Components | Monitor and keep track of changes in ecosystem parties, so that business processes are not interrupted or hide risks. |
| User instructions/Communication | | |
|  | User instructions | The instruction for use should provide information on the risk assessment for the device as regards to IT security risks |
|  | User instructions | The instruction could include high level summary of risk profile of the medical device and the corresponding IT security objectives |
|  | User instructions | The instruction could include Specifications of the operating system |
|  | User instructions | The instruction should include information about the installation, configuration and operation of the medical device |
|  | Security by default | Security configuration options; in accordance with the security-by-default principle, the medical device should have the highest possible security settings selected by default. |
|  | User instructions | The instruction could include Product installation |
|  | User instructions | The instruction could include Initial configuration guidelines, e.g. change of default passwords during first login. |
|  | User instructions | The instruction could include Step-by-step instructions for deploying security updates |
|  | User instructions | The instruction could include Procedures for using the medical device in failsafe mode (e.g. enter/exit failsafe mode, performance restrictions in failsafe mode, data recovery function when resuming normal operation etc.) |
|  | User instructions | The user instruction should include information about the secure configuration and application of security updates for the medical device |
|  | User instructions | The user instruction should outline compatibility issues as regards to the operating environment (software, hardware etc.) and any compatibility restrictions |
|  | User instructions | The user instruction should adequately describe the requirements regarding the operating environment (hardware, network characteristics, security controls etc.) |
|  | User instructions | The instruction could include Assumptions on the environment of use (e.g. home environment, healthcare facility etc.) |
|  | User instructions | A description of backup and restore features for both data and configuration settings |
|  | User instructions | Device instructions for use and product specifications related to recommended cybersecurity controls appropriate for the intended use environment (e.g., anti-virus software, use of a firewall, etc.). |
|  | User instructions | Description of device features that protect critical functionality, even when the device’s cybersecurity has been compromised (e.g. Operating System hardening). |
|  | User instructions | Description of backup and restore features and procedures to regain configurations. |
|  | User instructions | Specific guidance to users regarding supporting infrastructure requirements so that the device can operate as intended. |
|  | Information for providers | Description of how the device is or can be hardened using secure configuration. Secure configurations may include end point protections such as anti-malware, firewall/firewall rules, whitelisting, security event parameters, logging parameters, physical security detection, etc. |
|  | Information for providers | List of network ports and other interfaces that are expected to receive/send data, and a description of port functionality and whether the ports are incoming or outgoing (Unused ports should be disabled). |
|  | Information for providers | Sufficiently detailed network diagrams for end-users. |
|  | Information for providers | Where appropriate, technical instructions to permit secure network (connected) deployment and servicing, and instructions for users on how to respond upon detection of a cybersecurity vulnerability or incident. |
|  | Information for providers | Where appropriate, risks of using the medical device outside of the intended use environment. |
|  | User instructions | Recommended IT security controls for operating environment (e.g. anti-virus, firewall) |
|  | Up-to-date User instructions | The Manufacturer should update the instructions if needed |
|  | Information for providers | The MDS2 is an industry-wide and globally accepted form, which can be used to provide the abovementioned security information. |
|  | Tailored user instructions | Provision of information for Medical Device Software (MDSW) users should be tailored to where the device is used |
|  | Turn off features | User information should inform users to be able to Turn of features that are not used |
|  | Minimum requirements | nstruction should contain Minimum requirements for the workstations intended for user operations: hardware features, operating system versions, peripheral devices, etc. |
|  | Minimum requirements | Instruction should contain Minimum platform requirements for the connected medical device: hardware properties, operating system versions, middleware and drivers, peripheral devices, etc |
|  | Information for operators | Information to operators of medical devices on the identified risk and possible mitigations in the operating environment |
|  | User instructions | Instructions should contain Risks for device operation outside the intended operating environment |
|  | User instructions | Clinicians/physicians should be provided with the information they need to have meaningful discussion with their patients about the risks and benefits of the device they use, including cybersecurity risks. |
| Documentation | | |
|  | Up-to-date Security informations | Security information should be kept up-to-date |
|  | Documentation | technical documentation should include security requirements to ensure safety and effectiveness of products against security risks and threats, and a justification, validation and verification of the solutions adopted to meet those requirements |
|  | Up-to-date documentation | technical documentation needs to be updated with information raised through the manufacturers post market surveillance system related to handling and remediation of cybersecurity incidents and vulnerabilities |
|  | User instructions | The manufacturer shall provide clear documentation of the device’s instructions for use, including IT security features/configurations |
|  | Security Guidelines | The manufacturer should create and maintain security guidelines |
|  | SBOM | Software Bill of Materials for security information sharing |

Table S4. Comparison of the created cybersecurity requirements list with FDA_Cyber and MDCG 2019-16. The coverage of the themes is provided on a three-point Likert Scale from “Sufficiently covered” (Green, *) to “Partially covered” (Yellow, †) to “Insufficiently covered” (Red, ‡).

|  | Aspect | FDA_Cyber | MDCG 2019-16 |
| --- | --- | --- | --- |
| 01_General Principles | | * | * |
| GNRL_1 | Life-Cycle Approach | * | * |
| GNRL_2 | Security by Design (SbD) | * | * |
| GNRL_3 | Security by Default | * | * |
| GNRL_4 | Usable Security | ‡ | † |
| GNRL_5 | No Security through Obscurity | * | ‡ |
| GNRL_6 | Security in Context | * | * |
| GNRL_7 | Third Party Auditing | ‡ | * |
| GNRL_8 | End-Of-Life | ‡ | ‡ |
| GNRL_9 | Documentation | * | * |
| GNRL_10 | Manufacturing / Supply Chain | * | † |
| GNRL_11 | Secure Provisioning | † | ‡ |
| 02_Auth & Access Control | | † | ‡ |
| AUTH_1 | Principle of Least Privileges | * | * |
| AUTH_2 | Role Based Access Control | † | * |
| AUTH_3 | Multi-Factor Authentication (MFA) | * | ‡ |
| AUTH_4 | Session Management | * | * |
| AUTH_5 | Re-authentication after inactivity or interruptions | ‡ | ‡ |
| AUTH_6 | Access Logging | * | ‡ |
| AUTH_7 | Unusual login attempts | ‡ | † |
| AUTH_8 | Root of trust | † | ‡ |
| 03_Data Protection & Privacy | | † | † |
| DATA_1 | Privacy-preserving design | ‡ | ‡ |
| DATA_2 | Minimal access to sensitive data | ‡ | ‡ |
| DATA_3 | Secure Data at Rest | * | * |
| DATA_4 | Secure Data at Transit | * | * |
| DATA_5 | Secure Data at Use | ‡ | ‡ |
| DATA_6 | Third-party data access | ‡ | ‡ |
| DATA_7 | Data Minimisation | ‡ | ‡ |
| DATA_8 | Verifying data validity | * | * |
| 04_Cryptography | | * | ‡ |
| CRYP_1 | Strong Cryptographic Algorithms | * | † |
| CRYP_2 | Key Management | † | ‡ |
| CRYP_3 | Strong Cryptographic Keys | * | ‡ |
| CRYP_4 | Protection of cryptographic keys | ‡ | ‡ |
| CRYP_5 | No hard-coded keys, credentials or other secrets | * | ‡ |
| CRYP_6 | Proven Implementations | * | ‡ |
| 05_Architecture | | * | † |
| ARCH_1 | Secure Update Mechanism | * | * |
| ARCH_2 | Central logging system | * | * |
| ARCH_3 | Endpoint security | * | * |
| ARCH_4 | Management interfaces | ‡ | ‡ |
| ARCH_5 | Defense in Depth | * | * |
| ARCH_6 | Unique IDs | * | * |
| ARCH_7 | Zero trust architectures | † | ‡ |
| ARCH_8 | Design/Architecture reviews | * | ‡ |
| 06_Network | | † | * |
| NETW_1 | Network Security | † | * |
| NETW_2 | Firewalls | † | * |
| 07_Passwords | | * | * |
| PWRD_1 | Strong Password Rules | * | * |
| 08_Resilience | | † | * |
| RESI_1 | Integrity checks of components | * | * |
| RESI_2 | Redundancy | ‡ | ‡ |
| RESI_3 | Physical Security | * | * |
| RESI_4 | Fail-Safes | † | * |
| RESI_5 | Measures against Reverse engineering | ‡ | ‡ |
| 09_Source Code/Software Development | | * | ‡ |
| SOUR_1 | Coding Best Practises | * | ‡ |
| SOUR_2 | Input sanitation | * | ‡ |
| SOUR_3 | Secure development environment | * | ‡ |
| 10_Risk Management | | * | * |
| RISK_1 | Continuous Monitoring | * | * |
| RISK_2 | Incident Response Plan | * | * |
| RISK_3 | Backups | ‡ | * |
| RISK_4 | Threat Modeling | * | * |
| RISK_5 | Check vulnerability databases | * | * |
| RISK_6 | User instructions | * | * |
| RISK_7 | Quality Management System | ‡ | ‡ |
| RISK_8 | Security Training | * | * |
| RISK_9 | Security Risk Management | * | * |
| RISK_10 | User reporting | * | * |
| RISK_11 | Reporting of security-related issues to authorities | * | * |
| RISK_12 | Benefit Risk analysis | * | * |
| 11_Testing | | * | * |
| TEST_1 | Penetration testing | * | * |
| TEST_2 | Security requirements testing | * | * |
| TEST_3 | Threat mitigation testing | * | * |
| TEST_4 | Vulnerability testing | * | * |
| TEST_5 | Static code analysis | * | * |
| 12_Third Party Components | | † | * |
| THRD_1 | Limitation of third-party componenets | ‡ | * |
| THRD_2 | Maintenance of third-party components | * | † |
| THRD_3 | (S)BOM | * | * |
| THRD_4 | Secure third-party components/software | ‡ | * |
